# A role for EMT in CD73 regulation in breast cancer

**DOI:** 10.1080/2162402X.2022.2152636

**Published:** 2022-11-30

**Authors:** Meriem Hasmim, Guy Berchem, Bassam Janji

**Affiliations:** a Tumor Immunotherapy and Microenvironment Group, Department of Cancer Research, Luxembourg Institute of Health (LIH), Luxembourg City, Luxembourg; b Department of Hemato-Oncology, Centre Hospitalier du Luxembourg, Luxembourg City, Luxembourg

**Keywords:** CD73, SNAI1, epithelial-to-mesenchymal transition, adenosine, triple negative breast cancer, immunotherapy

## Abstract

CD73 is an emerging target in cancer due to its role in generating adenosine, a potent immunosuppressor. We found that SNAI1, a driver of epithelial-to-mesenchymal transition (EMT), upregulates CD73 in triple negative breast cancer cells. Here, we discuss the relevance of improving CD73-based therapy by combining with inhibitors of EMT.

Triple negative breast cancer (TNBC) subtype is devoid of estrogen receptors, progesterone receptors, and human epidermal growth factor receptor-2/neu (HER-2). TNBC represents 15% of all breast cancers and has the highest mortality rate and probability of disease relapse.

Conventional chemotherapy is still the only established therapeutic option to treat TNBC with unfortunately a high rate of recurrence, resistance, and metastases.^1^ Recent clinical trials in TNBC patients using immune checkpoint blockade resulted in a promising but still insufficient survival benefit, highlighting the need for alternative approaches.

CD39 and CD73 are cell-surface ectonucleotidases playing a major role in immunosuppression. Such a role relies on the sequential hydrolysis of extracellular adenosine triphosphate (ATP) into ADP and AMP by CD39, and the subsequent conversion of AMP into the broadly immunosuppressive molecule adenosine by CD73. Extracellular adenosine exerts its immunosuppressive functions by binding to its high and low affinity receptors A2A and A2B, respectively, on immune cells.^2^ This sequential action of CD39 and CD73 scavenges ATP from the tumor and increases extracellular adenosine, hence generating an immunosuppressive tumor microenvironment. Due to the critical role of CD73 in adenosine release, therapies based on CD73 targeting are currently under intense investigation for cancer treatment.

Although the expression of CD73 in TNBC tumor cells and other cancer types is generally associated with decreased overall and disease-free survivals,^3^ the molecular mechanisms responsible for CD73 regulation are still being uncovered. CD73 is described to be directly regulated by hypoxia-inducible factor-1 alpha (HIF-1α), a major transcription factor responsible for cellular adaptation to the hypoxic microenvironment. Considering that HIF-1 is also a potent inducer of epithelial-to-mesenchymal transition (EMT) transcription factors (EMT-TF) such as SNAIL1 and ZEB1,^4^ it is tempting to speculate that the EMT process can still regulate CD73 independently of hypoxia.

EMT is a well-established process activated in tumor cells to acquire aggressiveness and metastatic properties as well as therapy resistance.^5^ SNAI1 and ZEB1 are master regulators of EMT via promoter repression or activation of several genes associated with epithelial and mesenchymal phenotypes. We have previously reported that the EMT-TFs SNAI1 and ZEB1 are involved in the direct overexpression of the programmed cell death protein ligand-1 (PD-L1) and the macrophage immune checkpoint CD47 on tumor cells.^6,7^

In our recent publication,^8^ we show that during the activation of the EMT process, SNAI1 directly induces CD73 expression in human TNBC cells via direct binding to the proximal E-box motif in the CD73 promoter. Targeting SNAI1 resulted in a significant decrease in CD73 expression in mesenchymal TNBC cells. We found that the functional impact of SNAI1-dependent upregulation of CD73 is the release of sufficient amounts of extracellular adenosine to impair natural killer (NK) cell cytotoxicity and proliferation (Figure 1). Of note, the adenosine inhibitory effects on NK cells were time- and dose-dependent. We believe that SNAI1 is not the only EMT-TF involved in CD73 overexpression. This statement is supported by our meta-analysis data performed on 258 TNBC patients from the METABRIC dataset showing that, in addition to SNAI1, a positive correlation between *NT5E/*CD73 and *ZEB1* mRNAs was observed. This indicates that several EMT-TF can be involved in CD73 overexpression.

**Figure 1.**
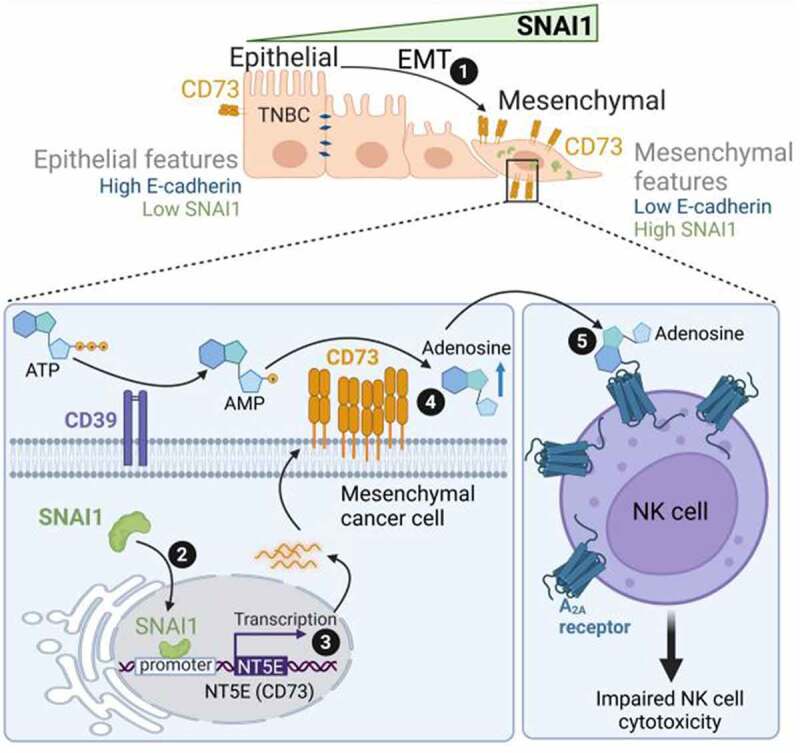
SNAI1-dependent EMT upregulates the expression of CD73 in TNBC cells. 1: Tumor cells undergoing EMT lose their epithelial features and gain mesenchymal properties characterized by high expression of the EMT-TF SNAI1. 2 and 3: SNAI1 translocates to the nucleus, binds to the proximal E-box motif of *NT5E/*CD73 gene to induce its expression. 4: Mesenchymal cells overexpressing CD73 release high amounts of the immunosuppressive molecule adenosine from adenosine monophosphate (AMP) in the tumor microenvironment of TNBC. 5: Through its binding to the high affinity receptor A_2A_, expressed on NK cells, adenosine impairs the cytotoxic function of these cells and contributes to the establishment of an immunosuppressive tumor microenvironment.

The EMT score, established based on the transcriptomic expression of several EMT markers in cancer, is a valuable tool allowing more objective estimations of the EMT phenotypes of tumors and cancer cell lines.^9^ The analysis of *NT5E/*CD73 mRNA expression in a large panel of TNBC cell lines shows a positive correlation between their EMT score and CD73 expression. These results support the concept that not only SNAI1 and ZEB1, but the EMT as a whole process is involved in the regulation of CD73.

Several preclinical studies demonstrated the efficacy of targeting CD73 pathway to sensitize resistant tumors to chemotherapy and immunotherapy. Moreover, a large number of clinical trials using CD73 inhibitors are undergoing to enhance the clinical benefit of immunotherapy and chemotherapy. Overall, our work highlights the contribution of EMT in CD73 transcriptional regulation in TNBC cells and supports the concept of evaluating the relevance of combining inhibitors of the EMT process to improve CD73- and other immune checkpoints-based cancer immunotherapy.

Although inhibiting CD73 as a strategy for cancer treatment is gaining major interest, the clinical use of such inhibitors should be carefully evaluated. Indeed, *NT5E*/CD73 is ubiquitously expressed and regulates critical homeostatic functions across multiple organs. In this regard, a recent review reflects on the risk of increased toxicity and organ injury related to the combination of CD73 inhibitors with other immune checkpoint blockades.^10^ We therefore believe that combining molecules inhibiting the EMT process can provide an opportunity to potentiate CD73 inhibitors, decrease their dosing regimens, and enlarge their use in combination with immunotherapy.

## Author’s view to

Hasmim M; Xiao M; Van Moer K, Kumar A; Oniga A; Mittelbronn M; Duhem C; Chammout A; Berchem G; Thiery JP; Volpert M; Hollier B; Noman MZ and Janji B. SNAI1-dependent upregulation of CD73 increases extracellular adenosine release to mediate immune suppression in TNBC. *Frontiers in Immunology*, Sep 9; 13:982821. doi: 10.3389/fimmu.2022.982821. eCollection (2022).

## Data sharing statement

This is an author’s view manuscript. Data availability statement is not applicable.
